# Mechanisms of Tetramycin-Induced Resistance to Rice Blast Disease in *Oryza sativa* L.

**DOI:** 10.3390/ijms27021024

**Published:** 2026-01-20

**Authors:** Hui Jiang, Caixia Zhao, Danting Li, Kai Sun, Yipeng Xu, Kun Pang, Xiaoping Yu, Xuping Shentu

**Affiliations:** Key Laboratory of Microbiological Metrology, Measurement & Bio-Product Quality Security, State Administration for Market Regulation, College of Life Science, China Jiliang University, Hangzhou 310018, China

**Keywords:** tetramycin, *Magnaporthe oryzae*, RNA sequence, enzyme activity, phytohormone, rice blast resistance

## Abstract

Rice blast, caused by the fungus *Magnaporthe oryzae*, is a devastating disease that threatens global food security, causing annual yield losses of 10–30%. Consequently, novel control strategies beyond conventional fungicides are urgently needed. Tetramycin, a polyene macrolide antibiotic, is known for its broad-spectrum antifungal activity. However, the specific mechanisms underlying its efficacy against rice blast remain to be fully elucidated. In this study, we demonstrate that tetramycin confers resistance through a dual mode of action. First, in vitro assays revealed that tetramycin directly inhibits *M. oryzae* mycelial growth. Second, and more critically, it functions as a potent immune elicitor in *Oryza sativa*. Transcriptome analysis coupled with physiological assays showed that tetramycin treatment triggers a rapid oxidative burst, characterized by significantly elevated activities of key defense enzymes, including superoxide dismutase, peroxidase, phenylalanine ammonia lyase, and polyphenol oxidase (PPO). This oxidative response is further orchestrated through the simultaneous activation of the jasmonic acid (JA) and salicylic acid (SA) signaling pathways, as evidenced by the distinct upregulation of their respective biosynthetic genes and hormone levels. Collectively, these findings indicate that tetramycin not only acts as a direct fungicide but also primes the rice innate immune system via a synergistic reactive oxygen species-JA-SA signaling network, offering a sustainable strategy for rice blast management.

## 1. Introduction

Rice (*Oryza sativa* L.) is a major global crop and a staple food for more than half of the world’s population [1]. However, it is subjected to numerous stresses, including abiotic and biotic stresses, throughout its growth cycle, including both abiotic and biotic stresses [2]. Among these stresses, rice blast, caused by the filamentous ascomycete *Magnaporthe oryzae*, is one of the most devastating diseases affecting rice production [3].

Rice blast can manifest at various growth stages of rice, infecting leaves, internodes, and panicle necks [4], It leads to substantial annual yield losses that typically range from 10% to 30% on a global scale, with the potential for total crop failure in severe outbreak years [5,6]. Current management relies mainly on the application of fungicides and the cultivation of resistant rice varieties [7]. However, the extensive use of fungicides poses several challenges, including adverse effects on beneficial microorganisms [8], accelerated pathogen resistance development, and environmental contamination such as residual toxicity in grains, soil degradation, and ecosystem disruption [9]. Moreover, the high mutation rate of *M. oryzae* frequently leads to the breakdown of resistance in rice cultivars, further complicating disease management in agricultural production [10]. Given these limitations, exploring sustainable, environmentally friendly strategies to enhance rice blast resistance is urgently needed. Consequently, this pursuit has become a major focus of contemporary research.

Tetramycin is a novel 26-membered tetraene macrolide antibiotic produced by *Streptomyces* var. Beijing. Its molecular structure comprises two primary bioactive components, namely, tetramycins A and B [11,12]. As a broad-spectrum agricultural antibiotic, tetramycin exhibits potent inhibitory activity against various plant pathogens. These pathogens include *M. oryzae* (the causal agent of rice blast), *Botrytis cinerea*, *Colletotrichum* spp. (the causal agents of anthracnose), *Phytophthora capsici* (the causal agent of pepper phytophthora), and *Penicillium* spp. (the causal agents of yellow mold) [13,14].

Notably, growing evidence indicates that certain antibiotics employ a dual mode of action in plant disease control: beyond direct antimicrobial effects, they can enhance plant immunity, thereby conferring induced resistance. Early studies identified R-5 a bioactive strain isolated from azalea. This strain produces actinomycete-derived polyene antibiotics that induce systemic resistance in plants [15]. Subsequent research demonstrated that in cucumber, metabolites from strain HD-087 considerably enhance host resistance to *Fusarium oxysporum* by upregulating the activities of key defense-related enzymes, including peroxidase (POD), phenylalanine ammonia lyase (PAL), and β-1,3-glucanase [16]. Furthermore, studies have shown that the combined application of tetramycin with tebuconazole and myclobutanil can synergistically activate the defense enzyme system in *Pseudostellaria heterophylla*. This activation enhances nutrient transport activity and markedly improves disease resistance [17]. Collectively these findings provide a theoretical foundation for the potential application of tetramycin in inducing plant immunity. However, whether tetramycin can effectively induce resistance in rice against blast disease, which is caused by *M oryzae*, remains unclear and has not been systematically investigated. Therefore, our present study aims to elucidate the role and mechanism underlying tetramycin-induced resistance in rice against blast disease, thereby providing a basis for its improved application in agricultural practices.

## 2. Results

### 2.1. Tetramycin Reduces Rice Blast Development

**Figure 1:**
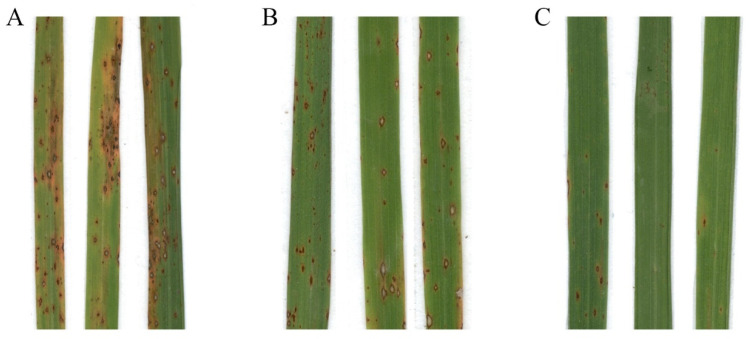
Tetramycin treatment reduces rice blast development. Blast infection assay of 4-week-old CO39 (*Oryza sativa* ssp. indica cultivar) seedlings with indicated concentrations of tetramycin. After spray inoculation, the rice seedlings were maintained in darkness for 24 h, then transferred to 12 h light/12 h dark for 6 days. Compared with control (**A**), blast development was suppressed significantly by treatment of tetramycin at concentrations of 3.84 (**B**) and 7.68 (**C**) mg/L. Samples with three replicates were prepared from these three groups.

Our previous research has shown that tetramycin has a strong inhibitory effect on the rice blast fungus [18]. Tetramycin exhibits strong inhibitory activity against *M. oryzae* and enhances rice blast resistance by activating defense-related enzymes, thereby preventing pathogen invasion. We treated four-week-old rice seedlings with tetramycin by using a foliar spray method to evaluate its effect against rice blast. The relative lesion areas upon treatment with tetramycin at concentrations of 3.84 and 7.68 mg/L were 30.99 ± 1.13 (n = 10, *p* < 0.05) and 5.47 ± 0.37 (n = 10, *p* < 0.05), respectively, reducing by 49% and 91%, respectively, compared with the lesions under the control treatment (60.76 ± 0.72, n = 10) (Figure 1).

### 2.2. Transcriptome Analysis of Global Gene Regulation by Tetramycin

We treated rice plants inoculated with *M. oryzae* with tetramycin at 24 hpi and then used leaf samples for subsequent RNA sequencing experiments at 30, 36, and 48 hpi to investigate the mechanisms of action of tetramycin in the induction of host defense responses during early *M. oryzae* infection. We utilized the rice leaves from the inoculation control group (without tetramycin) and experimental group treated with 7.68 mg/L tetramycin to obtain transcriptional profiles. We prepared samples with three biological replicates from these three groups.

**Figure 2:**
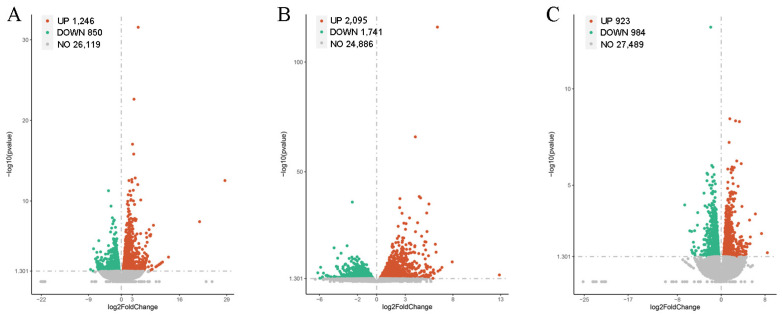
Volcano-plot of DEGs from the treatment group compared with the control at 30 (**A**), 36 (**B**) and 48 hpi (**C**), respectively. Red- and green-colored splashes represent significantly upregulated and downregulated genes. Grade-colored splashes represent genes without significant differences in their expression levels.

Deep RNA sequencing generated 4.50 × 10^7^, 3.95 × 10^7^, and 4.64 × 10^7^ valid reads for the experimental group at 30, 36, and 48 hpi, respectively, and 4.41 × 10^7^, 4.37 × 10^7^, and 4.45 × 10^7^ valid reads for the inoculation control at 30, 36, and 48 hpi, respectively. We mapped these sequence reads to the genome of *O*. *sativa*, resulting in the identification of 41,655 genes. Comparative transcriptome analysis between the expression profiles of the control and experimental groups revealed 1246 and 850 transcripts that were upregulated and downregulated at 30 hpi, respectively; 2095 and 1741 transcripts that were upregulated and downregulated at 36 hpi, respectively; and 923 and 984 transcripts that were upregulated and downregulated at 36 hpi, respectively (Figure 2).

**Figure 3:**
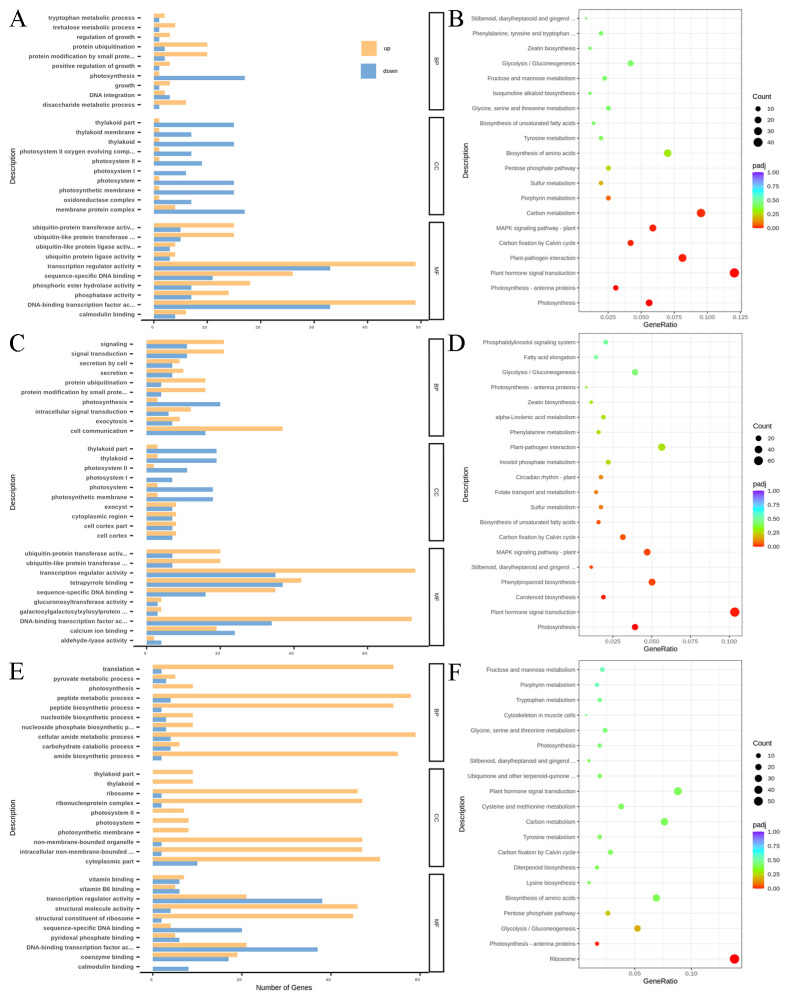
Enrichment analyses of DEGs. (**A**,**C**,**E**) GO classification of DEGs between the treatment group compared with the control at 30, 36 and 48 hpi, respectively. Along the vertical axis is the enriched GO term, and on the horizontal axis is the number of DEGs in a given term. Yellow- and blue-colored bars represent upregulated and downregulated genes, respectively. (**B**,**D**,**F**) KEGG pathway enrichment analysis of DEGs in the group between the treatment compared with the control at 30, 36 and 48 hpi, respectively. The ordinate represents the pathway name, the abscissa represents the rich factor, and the point size represents the number of DEGs in that pathway, while the point color denotes the differing Q-value ranges.

We performed Gene Ontology (GO) enrichment analysis for the functional annotation of differentially expressed genes (DEGs), which were classified into three main categories: molecular function (MF), biological process (BP), and cellular component (CC). Unigenes were categorized into different GO terms, with most DEGs belonging to MF in the groups between the control and treatment groups at 30 and 36 hpi belonging to MF (Figure 3A,B). A large number of DEGs were categorized into the MF categories transcription regulator activity and DNA-binding transcription activity. DEGs that were upregulated at 48 hpi were predominantly associated with BP and CC categories, whereas those that were downregulated at the same time point were mainly enriched the MF category (Figure 3C). In the BP category, the top five enriched terms associated with upregulated transcripts were translation, peptide metabolic process, peptide biosynthetic process, cellular amide metabolic process, and amide biosynthetic process. In the CC category, ribosome, ribonucleoprotein complex, nonmembrane-bounded, intracellular nonmembrane-bounded, and cytoplasmic part were the top five classes enriched in upregulated transcripts. In the MF category, transcription regulator activity, DNA-binding transcription activity and sequence-specific DNA-binding were the top three classes enriched in downregulated transcripts.

As shown in Figure 3B,D, KEGG pathway enrichment analysis revealed the significant enrichment of DEGs in several pathways when we compared the treatment and control groups at 30 and 36 hpi. Pathways included plant hormone signal transduction, the plant MAPK signaling pathway, carbon fixation in photosynthetic organisms, and photosynthesis. Further molecular characterization identified several key regulators in these pathways, including the genes of phytosulfokine receptor and auxin-responsive protein in the plant hormone signal transduction pathway, and LRR receptor-like serine/threonine-protein kinase and abscisic acid receptor in the MAPK cascade. At 48 hpi, DEGs involved in the ribosome and photosynthesis—antenna proteins pathways were significantly enriched in the treatment group relative to those in the control (Figure 3F). Notably upregulated genes included those encoding ribosomal proteins and light-harvesting complex proteins. Generally, genes with corrected *p* < 0.05 could be considered as enriched items.

### 2.3. Tetramycin Enhances Rice Resistance Against M. oryzae by Influencing Enzyme Activity

**Figure 4:**
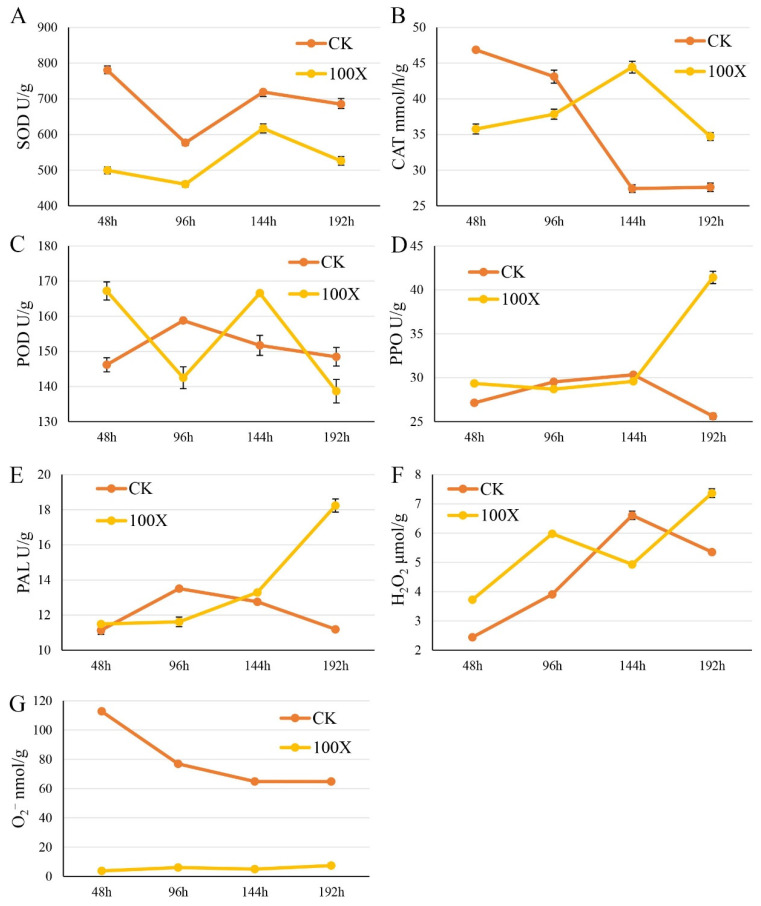
The effects of tetramycin on SOD (**A**), CAT (**B**), POD (**C**), PPO (**D**), PAL (**E**), H_2_O_2_ (**F**) and O_2_^−^ (**G**) activity in rice leaves. Enzymes activities between different groups were statistically analyzed by using Student’s *t*-test. *p* < 0.05 was considered to be statistically significant.

We measured the effects of tetramycin on the activities of key antioxidant enzymes and the levels of hydrogen peroxide (H_2_O_2_) and superoxide anion (O^2−^) in rice leaves to validate the effect of tetramycin solution on immune-related proteases in rice further (Figure 4). Analyses revealed the multifaceted role of tetramycin in modulating the plant immune defense system during pathogenic fungal infection. O^2−^ content sharply increased to a peak (approximately 120 nmol/g), in the treatment group at 48 h after tetramycin treatment and was several times higher than that in the control group, indicating the successful activation of a reactive oxygen species (ROS) burst. Concurrently, the activity of SOD in the treatment group also peaked at approximately 900 U/g, significantly exceeding that in the CK group, reflecting a prompt response to oxidative stress. Subsequently, the H_2_O_2_ content in the treatment group peaked at 96 h (approximately 100 μmol/g), and was markedly higher than that in the control group. This result indicates that as a key signaling molecule, H_2_O_2_ continued to regulate the expression of downstream defense genes. From 96 h to 192 h posttreatment, the phenylpropanoid metabolism pathway was strongly activated: the PAL activity in the treatment group increased continuously from 48 h to 192 h (from 12 U/g to 20 U/g), steadily remaining higher than that in the control group. The activity of PPO, a downstream enzyme, in the treatment group showed a similar upward trend (from 25 U/g to 45 U/g) and was significantly elevated compared with that in to the control group. Meanwhile, the activity of POD in the treatment group rose steadily (from 130 U/g to 170 U/g), significantly surpassing that in the control group. By contrast, the activity of catalase (CAT) decreased consistently throughout the experimental period (from 50 U/g to 25 U/g) and remained lower than that in the control group, a change that likely helps maintain the signaling function of intracellular H_2_O_2_. Collectively, these findings further reveal that tetramycin not only aids in resisting pathogenic fungi, but also triggers a series of immune responses in rice plants.

### 2.4. Tetramycin Enhances Rice Resistance Against Blast by Influencing the Level of Hormones

**Figure 5:**
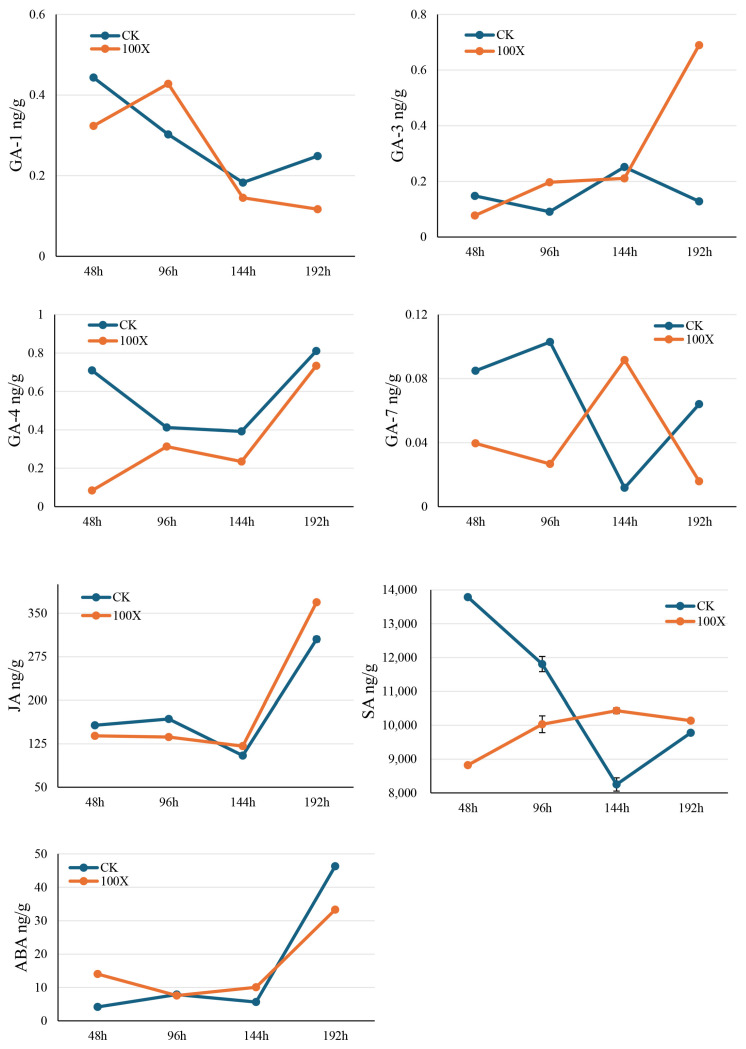
The effects of tetramycin on the content of different plant hormones in rice leaves. Hormone contents between different groups were statistically analyzed by using Student’s *t*-test. *p* < 0.05 was considered to be statistically significant.

**Figure 6:**
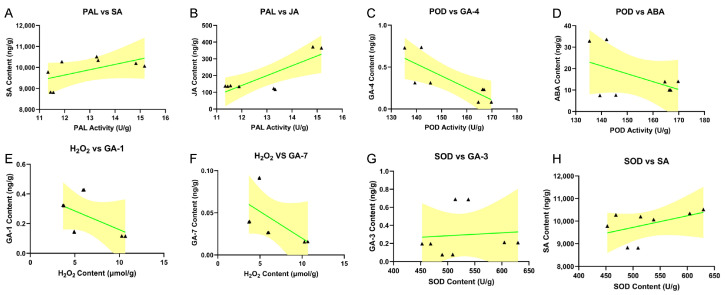
Pearson correlation analysis between individual enzyme activities and phytohormone contents. Scatter plots (**A**–**H**) show the relationships between (**A**) PAL activity and SA content, n = 8, r^2^ = 0.3356, *p* = 0.13. (**B**) PAL activity and JA content, n = 8, r^2^ = 0.6793, *p* = 0.01. (**C**) POD activity and GA-4 content, n = 8, r^2^ = 0.6435, *p* = 0.02. (**D**) POD activity and ABA content, n = 8, r^2^ = 0.2403, *p* = 0.22. (**E**) H_2_O_2_ content and GA-1 content, n = 8, r^2^ = 0.2531, *p* = 0.20. (**F**) H_2_O_2_ content and GA-7 content, n = 8, r^2^ = 0.3114, *p* = 0.15. (**G**) SOD activity and GA-3 content, and n = 8, r^2^ = 0.006657, *p* = 0.85. (**H**) SOD activity and SA content. n = 8, r^2^ = 0.2293, *p* = 0.23. Enzyme activities (PAL, POD, SOD) are expressed in units per gram fresh weight (U/g), H_2_O_2_ content in μmol per gram, and phytohormone levels in ng per gram fresh weight. Pearson’s correlation analysis was used to evaluate each relationship. A linear regression line is fitted to each scatter, and the shaded area around the line represents the 95% confidence interval of the regression. *p* < 0.05 is considered statistically significant. The green line represents the best-fit simple linear regression. The yellow shaded area indicates the 95% confidence band of the fitted regression line.

Concurrently, we also analyzed the spatiotemporal changes in the levels of seven key plant hormones—GA-1, GA-3, GA-4, GA-7, jasmonic acid (JA), salicylic acid (SA) and abscisic acid (ABA)—in rice leaves following tetramycin treatment (Figure 5). Comparative analysis across four time points (48, 96, 144, and 192 h) revealed distinct modulation patterns in hormone dynamics. Regarding gibberellins, tetramycin treatment generally suppressed the accumulation of GA-1 and GA-4. By contrast, it significantly promoted GA-3 biosynthesis at 144, and 192 h. The content of GA-7 in the treatment group also began to increase from 144 h onward, reaching a level that was more than 2-fold higher than that in the control group by 192 h. The JA content exhibited a steady increase after tetramycin application, with the JA content in the treatment group increasing by approximately 40% at 144 and 192 h relative to that in the control group, indicating the sustained activation of the JA signaling pathway. SA in the treatment group showed a pronounced response rising sharply between 96 and 144 h and eventually reaching more than 1.3 times that in the control group. The concurrent elevation of these defense-related hormones suggests that tetramycin may confer broad-spectrum resistance by activating JA and SA signaling pathways. Furthermore, ABA dynamics displayed a typical stress–response pattern. Its content in the treatment group rapidly increased by approximately 30% relative to that in the control group within 48–96 h posttreatment, reflecting an initial stress response. As the treatment progressed, ABA levels gradually declined and returned to the control level by 192 h, indicating a transition from an acute stress response to an adapted physiological state. We performed Pearson correlation analyses to bridge the observed enzymatic responses with phytohormone dynamics (Figure 6). PAL activity exhibited a positive association with JA and a similar trend with SA, consistent with the well-documented role of JA/SA signaling in activating the phenylpropanoid pathway and with PAL functioning as a key entry enzyme that can contribute to SA-related defense metabolism. In contrast, POD activity was negatively correlated with GA-4, supporting the concept of a growth–defense trade-off in which immune activation is commonly accompanied by reduced bioactive GA levels while cell-wall defense modules are reinforced. In addition, H_2_O_2_ displayed negative associations with GA-1 and GA-7, suggesting that oxidative signaling may coincide with attenuation of GA-mediated growth programs during the defense process. Notably, correlations involving ABA and antioxidant enzymes did not always reach statistical significance, which may reflect temporal phase differences between early hormonal signaling and subsequent redox-enzyme activation, as well as the limited sample size.

## 3. Discussion

Tetramycin, a polyene macrolide antibiotic produced by *Streptomyces* spp., exhibits broad-spectrum fungicidal activity [14,19]. Similarly to other *Streptomyces* metabolites, tetramycin provides dual protection: the direct suppression of pathogens via antimicrobial action and induction of host defenses through biochemical signaling [19,20]. In vitro and in vivo assays have shown that tetramycin strongly inhibits the growth, spore germination and appressorium formation of *M. oryzae* [14]. Biochemical studies on related pathogens have demonstrated that tetramycin causes membrane damage and ergosterol depletion, consistent with typical polyene antibiotic action [14]. Notably, tetramycin’s mode of action is considered unique and unlikely to engender pathogen resistance [19], a feature that distinguishes it from numerous conventional fungicides.

Beyond its direct antibiotic properties, tetramycin markedly enhances rice immune responses. Transcriptomic analysis revealed that tetramycin treatment substantially upregulates genes involved in plant hormone signal transduction and other defense-related pathways. This result aligns with the established paradigm of plant immunity, wherein chemical elicitors trigger complex signaling networks to activate defense mechanisms [21,22]. Notably, exogenous JA application alone is known to induce similar changes: In rice, JA application induces OsAOS2, OsLOX3, and OsJAZs along with the SA pathway regulators OsPR1a and OsWRKY45 and even upregulates the NADPH oxidase OsRbohB and peroxidase OsPOX1 [23]. The tetramycin-elicited increase in the activity of enzymes including PAL, POD, PPO and SOD, implies that rice plants perceive tetramycin as an elicitor, thereby activating inducible defense gene networks. Consistent with this mechanism, other actinomycete-derived elicitors, such as ningnanmycin, have been shown to boost PAL, POD, and SOD expression and SA biosynthesis, triggering systemic acquired resistance [24].

The analysis of phytohormone dynamics provides further insight into the immunomodulatory role of tetramycin in rice [6,25]. Previous studies have established JA as a crucial hormone for blast resistance. Rice mutants with JA biosynthesis deficiency exhibit enhanced susceptibility to *M. oryzae*, whereas those overexpressing JA-biosynthetic genes show promoted pathogenesis-related gene expression during infection [25]. In our study, tetramycin treatment led to marked increases in endogenous JA and JA–Ile levels, as well as the accumulation of SA at 48 h posttreatment. Interestingly, rice plants typically do not elevate SA levels upon *M. oryzae* attack; indeed, a defense response in rice usually requires exogenous SA application [6]. In this context, tetramycin’s effects resemble those of the synthetic SA analog BTH, which strongly activates SA-dependent defenses including WRKY45 and other PR genes [6]. However, in contrast to BTH, tetramycin simultaneously activates the JA pathway, as evidenced by the synergistic induction of JA-responsive transcripts together with SA markers. This JA–SA coactivation aligns with rice’s unique hormone interplay and is likely key to a broad-spectrum defense. While our study demonstrates that tetramycin activates JA and SA signaling pathways, the upstream sensors or receptors that perceive the tetramycin signal remain unknown. Future work should focus on identifying the direct molecular targets of tetramycin in rice cells to elucidate fully the mechanism by which it triggers these defense cascades.

A hallmark of plant immune activation is the oxidative burst. We detected a rapid burst of ROS in tetramycin-treated rice, including elevations of O^2−^ and H_2_O_2_. Early ROS production represents a common feature of pattern-triggered immunity. However, it is often described as atypical during *M. oryzae* infection [6]. ROS serve dual functions: at high concentrations, they can directly inhibit invading pathogens, whereas at controlled levels, they act as signaling molecules to activate downstream defense responses [6,26]. Our experiments revealed the strong upregulation of both ROS-scavenging and ROS-producing enzymes. SOD activity increased, consistent with the conversion of O^2−^ into H_2_O_2_. Similarly, POD and PPO activities rose markedly; these enzymes utilize H_2_O_2_ to catalyze lignin and phenolic cross-linking in the cell wall, reinforcing barriers against pathogen ingress. PAL activity also increased, feeding into the phenylpropanoid pathway that produces phytoalexins and SA. These changes mirror those seen with other immune elicitors: for example, the treatment of plants with *Streptomyces*-derived ningnanmycin or with chitosan considerably boosts PAL, POD, and SOD activities as part of induced resistance [6,26]. Collectively, the surge in ROS coupled with enhanced antioxidant enzyme activities suggests that tetramycin triggers a controlled redox signaling cascade and thus resembles classic elicitors of plant immunity.

By contrast, tetramycin integrates direct antimicrobial activity with plant immune activation. At low concentrations, tetramycin can eliminate *M. oryzae* inoculum and simultaneously vaccinate rice plants, an effect unattainable with BTH alone. Chitosan, an oligosaccharide elicitor, also induces SAR and defense enzymes [24]. However, it has a broad mode of action that is broader, encompassing microbial membrane disruption and nutrient chelation, thereby acting in part as a direct antimicrobial agent [24]. Nevertheless, the antimicrobial efficacy of chitosan is generally lower than that of specialized antibiotics. Quantitative analysis indicates that its antimicrobial activity is highly dependent on molecular weight, with optimal activity typically occurring within a critical range of 4–10 kDa [27]. Tetramycin’s well-defined chemical structure and potent bioactivity distinguish it from other elicitors. Furthermore, other actinomycete-derived bioactive compounds (e.g., aminoglycosides like kasugamycin, or lipopeptides like fengycins) have been shown to elicit defenses, but few have documented direct antifungal properties against rice blast coupled with immune induction. Tetramycin’s tetraene macrolide framework thus represents a distinctive mode: it targets the pathogen’s membrane integrity while also engaging the host’s JA/SA/ROS machinery for an amplified response.

Collectively, our findings reveal the mechanism underlying tetramycin-induced resistance in rice. This dual action—direct fungicidal activity and plant immunomodulation—creates a potent synergistic effect. Tetramycin directly targets *M. oryzae*, reducing the initial pathogen load. In addition, it primes rice innate immunity by triggering a rapid ROS burst, upregulating JA and SA pathways, and enhancing the activity of key defense enzymes (SOD, POD, PPO, and PAL). These changes result in the enhanced fortification of cell walls and accumulation of antimicrobial phenolics. The transcriptomic shifts we observed align with an early-wave defense reminiscent of effector-triggered immunity, albeit triggered here by a chemical agent rather than a pathogen effector. From a biological standpoint, these insights advance our understanding of how chemical elicitors can modulate rice blast resistance, highlighting potential for designing new inducers that exploit similar dual mechanisms.

## 4. Materials and Methods

### 4.1. Test Materials

The rice blast fungus (*M. oryzae*) used in this study was obtained from the Liaoning Microbial Strain Preservation Center. The rice cultivar employed was CO39 (*O. sativa* ssp *indica* cultivar). A 0.15% aqueous solution of tetramycin (Wuningmycin, PD20171878) supplied by Liaoning Weike Biotechnology Co., Ltd. (Shenyang, Liaoning, China) served as the chemical treatment.

### 4.2. Rice Cultivation and Treatment Conditions

Rice seedlings were established by using a hydroponic pot system and the nutrient solution recipe from the International Rice Research Institute (composition detailed in Table 1). Uniform seedlings (at the 4–5 leaf stage) were divided into two treatment groups with 30 plants per replicate (three replicates per treatment). Throughout the experiment, rice plants were maintained under the controlled conditions of 22 °C ± 1 °C, 90–95% relative humidity, and 16/8 h (light/dark) photoperiod. One group of plants received a foliar application of tetramycin (7.68 mg/L), whereas the control group was treated with an equivalent volume of sterile water. Both treatments were administered 48 h prior to inoculation. Following inoculation, all plants were placed in the dark for 24 h to facilitate pathogen infection. The in vitro antifungal activity of tetramycin against *M. oryzae* was determined by using the mycelial growth rate method. The half-maximal effective concentration was calculated to be 7.68 mg/L from the resulting dose–response curve.

### 4.3. Preparation of P. oryzae Conidial Suspension

For inoculum preparation, cryopreserved *P. oryzae* was first activated and cultured on oatmeal–tomato juice agar at 25–28 °C until a dense mycelial mat formed. Forty-eight hours before inoculation, the surface mycelia were gently removed by scraping and rinsing with sterile water. Cultures were subsequently incubated under high humidity and continuous light for 1–2 days to induce conidiation. A conidial suspension was obtained by washing the culture surface with 100 mL of sterile water, filtering through a double layer of sterile gauze, and adjusting the concentration to approximately 30 conidia per 120× microscopic field [18].

### 4.4. Inoculation, Incubation, and Sampling Procedures

Inoculation was conducted by uniformly spraying the conidial suspension onto the foliage of rice seedlings. Treatments were separated by plastic barriers to prevent cross-contamination. After inoculation, plants were maintained in a high-humidity environment (e.g., in a shaded growth chamber) for 24 h, then returned to standard growth conditions. Leaf samples (the fourth and fifth leaves) were collected at 48, 96, 144, and 192 hpi. All collected samples were immediately frozen at −20 °C until further analysis.

### 4.5. cDNA Library Construction, Sequencing, and Bioinformatics Analysis

Total RNA was extracted from rice leaves by using the RNAprep Pure Plant Plus Kit (TIANGEN, Beijing, China). Its integrity of the total RNA was then verified by employing an Agilent 2100 Bioanalyzer (Agilent Technologies, Santa Clara, CA, USA). After RNA quality was confirmed, Hieff NGS^®^ Ultima Dual-mode RNA Library Prep Kit (premixed version) (Yeasen, Shanghai, China) was utilized for transcriptome library construction, strictly following the instructions regarding recommended reagents and consumables. For sequencing on the DNBSEQ-T7 platform, double-stranded products from the cDNA were denatured, circularized, and digested to generate single-stranded circular cDNA, which was used to prepare DNA nanoballs. After the libraries are qualified, sequencing was performed on the DNBSEQ platform in accordance with effective library concentrations and data output requirements.

The raw sequencing reads contained low-quality and adapter-contaminated reads. Therefore, quality control was performed by using the default parameters of fastp (v0.23.2) to filter substandard reads. Clean reads were quickly and accurately aligned to the *O sativa* L. reference genome (FA download link: https://www.ncbi.nlm.nih.gov/datasets/genome/GCF_034140825.1/ (accessed on 20 July 2025); GFF3 download link: https://www.ncbi.nlm.nih.gov/datasets/gene/GCF_034140825.1/ (accessed on 20 July 2025)) by utilizing HISAT2 software to obtain information on the positioning of reads on the reference genome. The transcripts of each sample were assembled by employing StringTie software (http://ccb.jhu.edu/software/stringtie/index.shtml (accessed on 20 July 2025)), and then merged into a set of transcript sets. The Fragments Per Kilobase of transcript per Million fragments mapped method was applied to measure the expression of a gene or transcript by StringTie with the maximum flow algorithm. Criteria for DEGs were set as fold change ≥ 2 and FDR < 0.05. Finally, GO and KEGG enrichment analyses were performed on the DEGs identified in all groups (http://geneontology.org (accessed on 25 August 2025); http://www.genome.jp/kegg/ (accessed on 25 August 2025)).

### 4.6. Measurement of Physiological Parameters

The activities of SOD and POD were determined by using the nitroblue tetrazolium photochemical reduction method and guaiacol methods, respectively. The activities of CAT and PPO were assessed via the catalytic decomposition of hydrogen peroxide ammonium molybdate. PAL activity was determined by monitoring the increase in absorbance at 290 nm resulting from the formation of *trans*-cinnamic acid through L-phenylalanine deamination. H_2_O_2_ and O^2−^ contents were determined by utilizing the titanium sulfate (titanium chloride), hydroxylamine hydrochloride paminobenzene sulfonic acid-α-naphthylamine colorimetric method, and the thiobarbituric acid colorimetric methods. All physiological parameters were analyzed by Wuhan ProNets Testing Technology Co., Ltd., Wuhan, China. Related enzymes activities between different groups were statistically analyzed by using Student’s *t*-test. A *p* < 0.05 was considered to be statistically significant.

### 4.7. Assays of the Different Phytohormones in Rice Leaves

Rice leaf samples (200–500 mg) were collected and ground into a fine powder in liquid nitrogen. The contents of the phytohormones gibberellins (GA-1, GA-3, GA-4, and GA-7), JA, SA, and ABA were quantified by using high-performance liquid chromatography–tandem mass spectrometry with a SCIEX QTRAP 5500 system (AB Sciex, Framingham, MA, USA). The powdered sample was mixed with acetonitrile solution (*v*/*v*), which contained 20 ng of deuterium-labeled internal standard per sample, extracted at 4 °C overnight, then centrifuged at 12,000 rpm for 5 min. The resulting supernatant was collected. The precipitate was re-extracted with acetonitrile, and the resulting supernatant was combined with the first extract. The combined supernatant was loaded onto a C18 Sep-Pak solid-phase extraction column. The column was vortexed for 30 s, and the flow-through was collected. The purified extract was dried under a gentle nitrogen stream and reconstituted in 100% methanol. The final extract was transferred to HPLC vials and analyzed through liquid chromatography (LC)–mass spectrometry (MS) on a SCIEX-6500QTRAP LC/MS/MS system (AB Sciex, Framingham, MA, USA) equipped with an electrospray ionization (ESI) turbo ion-spray interface. The contents of all phytohormones were determined by Wuhan ProNets Testing Technology Co., Ltd., Wuhan, China Chromatographic separation was performed by using a Waters XSelect^®^ HSS T3 column (Waters Corporation, Milford, MA, USA). The mobile phase consisted of (A) 0.1% formic acid in water and (B) acetonitrile. The flow rate was set at 0.35 mL/min, and the column temperature was maintained at 30 °C. An injection volume of 5 μL was used for analysis. Separation was achieved by employing a gradient elution program. Analysis was conducted with an electrospray ionization (ESI) source operating in positive and negative ionization modes. The key parameters were set as follows: curtain gas of 35 psi; ion spray voltage of +4500 V/−4500 V; nebulizing gas pressure of 60 psi; auxiliary gas pressure of 60 psi; and turbo spray temperature of 500 °C). Hormone contents between different groups were statistically analyzed by using Student’s *t*-test. *p* < 0.05 was considered to be statistically significant.

## 5. Conclusions

Our study elucidates the dual mechanisms by which tetramycin confers resistance to rice blast disease. Our findings demonstrate that tetramycin functions not only as a potent fungicide that directly inhibits *M. oryzae* growth but also as an effective immune elicitor that primes host defense responses. This induced resistance is mediated through a rapid oxidative burst, the enhanced activity of defense-related enzymes (SOD, POD, PAL, and PPO), and the synergistic activation of the JA and SA signaling pathways. These complex physiological and transcriptomic reconfigurations markedly curb pathogen expansion in rice tissues. Future research should focus on identifying the specific molecular targets or receptors in rice that perceive tetramycin signals, as well as mapping the precise transcriptional regulatory networks involving key transcription factors, such as WRKYs. Additionally, long-term field trials are necessary to evaluate the stability of tetramycin-induced immunity under fluctuating environmental conditions. Furthermore, given its antibiotic nature, investigating the effect of tetramycin application on the structure and function of the beneficial rice rhizosphere microbiome will be crucial for optimizing its sustainable deployment in green agriculture.

## Figures and Tables

**Table 1:** Composition of the modified nutrient solution used for rice hydroponic culture.

Composition	1 L/g
NH_4_NO_3_	91.6
CaCl_2_	88.6
NaH_2_PO_4_ 2H_2_O	40.3
K_2_SO_4_	71.4
MgSO_4_ 7H_2_O	324
MnCl_2_ 4H_2_O	1.5
H_3_BO_3_	0.934
CuSO_4_ 5H_2_O	0.031
(NH_4_)Mo_7_O_24_ 4H_2_O	0.074
ZnSO_4_ 7H_2_O	0.035
FeCl_3_ 6H_2_O	7.7
C_6_H_8_O_7_ H_2_O	11.9
H_2_SO_4_	50 mL

The components listed were weighed and dissolved in distilled water to a final volume of 1 L.

## Data Availability

Data and materials can be obtained from the research group upon request.

## References

[1] Duan S., Ai H., Liu S., Zhou A., Cao Y., Huang X. (2024). Functional nutritional rice: Current progresses and future prospects. Front. Plant Sci..

[2] Qiu J., Chen Y., Liu Z., Wen H., Jiang N., Shi H., Kou Y. (2023). The application of zinc oxide nanoparticles: An effective strategy to protect rice from rice blast and abiotic stresses. Environ. Pollut..

[3] Dean R., Van Kan J.A., Pretorius Z.A., Hammond-Kosack K.E., Di Pietro A., Spanu P.D., Rudd J.J., Dickman M., Kahmann R., Ellis J. (2012). The Top 10 fungal pathogens in molecular plant pathology. Mol. Plant Pathol..

[4] Qi Z.Q., Pan X., Du Y., Shen L., Yu M.N., Cao H.J., Song T.Q., Yu J.J., Zhang R.S., Yong M.L. (2020). Pathogenicity and population structure analysis of *Pyricularia oryzae* in different districts of Jiangsu province, China. Plant Pathol..

[5] Skamnioti P., Gurr S.J. (2009). Against the grain: Safeguarding rice from rice blast disease. Trends Biotechnol..

[6] Devanna B.N., Jain P., Solanke A.U., Das A., Thakur S., Singh P.K., Kumari M., Dubey H., Jaswal R., Pawar D. (2022). Understanding the Dynamics of Blast Resistance in Rice-*Magnaporthe oryzae* Interactions. J. Fungi..

[7] Xiong Z.Q., Tu X.R., Wei S.J., Huang L., Li X.H., Lu H., Tu G.Q. (2013). In vitro antifungal activity of antifungalmycin 702, a new polyene macrolide antibiotic, against the rice blast fungus *Magnaporthe grisea*. Biotechnol. Lett..

[8] Almasri H., Tavares D.A., Pioz M., Sene D., Tchamitchian S., Cousin M., Brunet J.L., Belzunces L.P. (2020). Mixtures of an insecticide, a fungicide and a herbicide induce high toxicities and systemic physiological disturbances in winter *Apis mellifera* honey bees. Ecotoxicol. Environ. Saf..

[9] Komarek M., Cadkova E., Chrastny V., Bordas F., Bollinger J.C. (2010). Contamination of vineyard soils with fungicides: A review of environmental and toxicological aspects. Environ. Int..

[10] Yura W.F., Muhammad F.R., Mirza F.F., Maurend Y.L., Widyantoro W., Farida S.S., Aziz Y.P., Desti A., Edy W., Septy M. (2021). Pesticide residues in food and potential risk of health problems: A systematic literature review. IOP Conf. Ser. Earth Environ. Sci..

[11] Cao B., Yao F., Zheng X., Cui D., Shao Y., Zhu C., Deng Z., You D. (2012). Genome mining of the biosynthetic gene cluster of the polyene macrolide antibiotic tetramycin and characterization of a P450 monooxygenase involved in the hydroxylation of the tetramycin B polyol segment. Chembiochem.

[12] Ren J., Cui Y., Zhang F., Cui H., Ni X., Chen F., Li L., Xia H. (2014). Enhancement of nystatin production by redirecting precursor fluxes after disruption of the tetramycin gene from *Streptomyces ahygroscopicus*. Microbiol. Res..

[13] Ma D., Zhu J., He L., Cui K., Mu W., Liu F. (2018). Baseline Sensitivity and Control Efficacy of Tetramycin Against *Phytophthora capsici* Isolates in China. Plant Dis..

[14] Gao Y., He L., Li X., Lin J., Mu W., Liu F. (2018). Toxicity and biochemical action of the antibiotic fungicide tetramycin on Colletotrichum scovillei. Pestic. Biochem. Physiol..

[15] Shimizu M., Furumai T., Igarashi Y., Onaka H., Nishimura T., Yoshida R., Kunoh H. (2001). Association of induced disease resistance of rhododendron seedlings with inoculation of *Streptomyces* sp. R-5 and treatment with actinomycin D and amphotericin B to the tissue-culture medium. J. Antibiot..

[16] Zhao S., Du C.M., Tian C.Y. (2012). Suppression of *Fusarium oxysporum* and induced resistance of plants involved in the biocontrol of Cucumber Fusarium Wilt by *Streptomyces bikiniensis* HD-087. World J. Microbiol. Biotechnol..

[17] Tian B., Tang C., Liu J., Jin B., Zhang C. (2025). Tetramycin ameliorates tebuconazole.azoxystrobin to control leaf spot and viral diseases of Taizishen. Front. Plant Sci..

[18] Jin H., Jiang H., Li D., Xu Y., Sun K., Pang K., Yu X., Shentu X. (2025). Antifungal activity and mechanism of the antibiotic fungicide tetramycin against *Magnaporthe oryzae*. Front. Agron..

[19] Wang Q., Zhang C., Long Y., Wu X., Su Y., Lei Y., Ai Q. (2021). Bioactivity and Control Efficacy of the Novel Antibiotic Tetramycin against Various Kiwifruit Diseases. Antibiotics.

[20] Dow L., Gallart M., Ramarajan M., Law S.R., Thatcher L.F. (2023). *Streptomyces* and their specialised metabolites for phytopathogen control—Comparative in vitro and *in planta* metabolic approaches. Front. Plant Sci..

[21] Jones J.D., Dangl J.L. (2006). The plant immune system. Nature.

[22] Pieterse C.M., Van der Does D., Zamioudis C., Leon-Reyes A., Van Wees S.C. (2012). Hormonal modulation of plant immunity. Annu. Rev. Cell Dev. Biol..

[23] Yang D.-L., Yao J., Mei C.-S., Tong X.-H., Zeng L.-J., Li Q., Xiao L.-T., Sun T.-P., Li J., Deng X.-W. (2012). Plant hormone jasmonate prioritizes defense over growth by interfering with gibberellin signaling cascade. Proc. Natl. Acad. Sci. USA.

[24] Fan H., Yan X., Fu M., Liu D., Awan A.W., Chen P., Rasheed S.M., Gao L., Zhang R. (2022). Interactive Effect of Biological Agents Chitosan, Lentinan and Ningnanmycin on Papaya Ringspot Virus Resistance in Papaya (*Carica papaya* L.). Molecules.

[25] Ma J., Morel J.B., Riemann M., Nick P. (2022). Jasmonic acid contributes to rice resistance against *Magnaporthe oryzae*. BMC Plant Biol..

[26] Haghpanah M., Namdari A., Kaleji M.K., Nikbakht-Dehkordi A., Arzani A., Araniti F. (2025). Interplay Between ROS and Hormones in Plant Defense Against Pathogens. Plants.

[27] Másson M. (2024). The quantitative molecular weight-antimicrobial acivity relationship for chitosan polymers, oligomers, and derivatives. Carbohydr. Polym..

